# Correlation between GAL-3, Klotho, Calcium and Phosphorus Metabolism Indexes and Cardiovascular Complications in patients with Chronic Kidney Disease

**DOI:** 10.12669/pjms.39.4.6988

**Published:** 2023

**Authors:** Zhe Li, Jian-Long Li, Qian Wang, Xing Fan, Yan Gao, Xue-Zhong Li

**Affiliations:** 1 Zhe Li, Department of Nephrology, Affiliated Hospital of Hebei University, Baoding 071000, Hebei, China; 2 Jian-Long Li, Department of Cardiology, Affiliated Hospital of Hebei University, Baoding 071000, Hebei, China; 3 Qian Wang, Department of Nephrology, Affiliated Hospital of Hebei University, Baoding 071000, Hebei, China; 4 Xing Fan, Department of Nephrology, Affiliated Hospital of Hebei University, Baoding 071000, Hebei, China; 5 Yan Gao, Department of Nephrology, Affiliated Hospital of Hebei University, Baoding 071000, Hebei, China; 6 Xue-Zhong Li, Intensive Care Unit, Affiliated Hospital of Hebei University, Baoding 071000, Hebei, China

**Keywords:** Chronic kidney disease, Calcium, phosphorus, GAL-3, Klotho, Cardiovascular complications

## Abstract

**Objective:**

To investigate the correlation between GAL-3, Klotho, calcium and phosphorus indexes and cardiovascular complications in patients with chronic kidney disease (CKD).

**Methods:**

This is a retrospective study. Forty patients with CKD and cardiovascular complications admitted to the Affiliated Hospital of Hebei University from February 20, 2022 to February 20, 2023 were selected as the experimental group, and another 40 patients with CKD without cardiovascular complications were selected as the control group. The differences in serum Ca^+2^, PO^-^_4_, GAL-3 and Klotho levels between the two groups were analyzed, and the correlations between the above indicators levels and creatinine levels were analyzed. The correlation between the above indicators levels and cardiac function classification was analyzed, and analyzed the risk factors of CKD complicated with cardiovascular complications.

**Results:**

The levels of Ca^+2^, PO^-^_4_ and GAL-3 in the experimental group were significantly higher than those in the control group, while the level of Klotho was significantly lower than that in the control group. The levels of Ca^+2^ and PO^-^_4_ were positively correlated with the level of Creatinine (Cr), while the level of Klotho was negatively correlated with the Cr. The levels of Ca^+2^ and PO^-^_4_ were positively correlated with cardiac function classification, while the level of Klotho was negatively correlated with cardiac function classification. Logistic regression analysis showed that hypertension, BMI, Cr, Ca^+2^, PO^-^_4_ and VLDL were risk factors for cardiovascular complications, and Klotho level was a protective factor.

**Conclusion:**

A positive correlation can be seen between the levels of Ca^+2^, PO^-^_4_ and cardiac function classification in patients with CKD. Klotho is a protective factor for cardiovascular diseases.

## INTRODUCTION

Chronic kidney disease (CKD), a common clinical disease in nephrology, develops gradually from various chronic diseases. With the progress of the disease, renal function gradually decreases and eventually progresses to end-stage renal disease (ESRD).^1^,^2^ It was shown in a study^3^ that the majority of patients with CRF developed cardiovascular complications, and the occurrence and development of cardiovascular disease (CVD) is an important factor affecting the prognosis of patients with CRF.^4^ Cardiovascular complications are dominated by vascular disease, valvular disease, ischemic heart disease, cardiac dysfunction, myocardial interstitial fibrosis and left ventricular hypertrophy,^5^,^6^ among which atherosclerotic cardiovascular disease and heart failure are the most common.^7^ Data show^8^ that patients with CVD suffer from about 15 times higher mortality than the general population, which is a critical risk factor for poor prognosis of CRF.^9^,^10^

Clinical studies have shown that among CRF patients without renal transplantation or dialysis treatment, about 45% of them are accompanied by cardiovascular events such as heart failure and coronary heart disease. With the decline of renal function in patients, their incidence of cardiovascular events gradually increases.^11^ To this end, early recognition and intervention should be strengthened for CRF patients complicated with CVD, which are beneficial to ameliorate their prognosis. Sarnak et al.^12^ suggested that the main risk factors for CRF complicated with CVD include abnormal calcium and phosphorus metabolism, inflammation, oxidative stress status, GAL-3, and Klotho levels, in addition to the most common traditional ones such as diabetes and hypertension. In this study, the correlation between calcium and phosphorus metabolism indexes, galectin-3 (Gal-3) and soluble Klotho protein levels and cardiovascular complications in patients with CKD was analyzed, so as to provide a reference for the treatment of CKD.

## METHODS

### General data:

This is a retrospective study. Forty patients with CKD and cardiovascular complications admitted to the Affiliated Hospital of Hebei University from February 20, 2022 to February 20, 2023 were selected as the experimental group. Cardiovascular complications, including myocardial infarction, myocardial ischemia, arrhythmia, left ventricular enlargement, etc. were confirmed by ultrasonic cardiogram, electrocardiogram or coronary angiography. Another 40 patients with CKD without cardiovascular complications who were hospitalized during the same period were selected as the control group. In the experimental group, there were 27 males and 13 females, aged 62-75 years, with an average of 68.57±6.53 years. In the control group, there were 23 males and 17 females, aged 56-74 years, with an average of 67.72±6.25 years. No significant difference was observ

### Ethical Approval:

The study was approved by the Institutional Ethics Committee of Affiliated Hospital of Hebei University on February 15, 2023 (HDFYLL-KY-2023-018), and written informed consent was obtained from all participants.

### Inclusion criteria:

- Patients who met the diagnostic criteria for CKD; 13
- Patients < 75 years old;
- Patients with a complete medical history and high compliance;
- Patients who themselves or their family members agreed to the study protocol and signed the consent form.

### Exclusion criteria:

- Patients who had received renal replacement therapy;
- Patients with congenital heart disease;
- Patients with valvular heart disease;
- Patients with active rheumatic disease;
- Patients who had taken medications that affected the results of the study within three months prior to admission.

### Testing indexed and methods:

Four milliliter of venous blood samples were drawn from all the included cases in the early morning on an empty stomach, left standing for 20 minutes, centrifuged at 2000 r/ minutes, for 20 minutes, and then the supernatant was collected and stored in a refrigerator at -80°C. Biochemical indicators such as Cr, Ca^+2^, PO^-^_4_, HDL, LDL, and VLDL were detected by an automatic biochemical analyzer. Serum Klotho protein and GAL-3 were detected by enzyme-linked immunosorbent assay.

The differences in serum Ca^+2^, PO^-^_4_, GAL-3 and Klotho levels between the two groups were compared and analyzed, and the correlations between serum GAL-3, Klotho, calcium, phosphorus levels and creatinine levels were also analyzed. Moreover, the correlation between serum calcium, phosphorus, GAL-3, Klotho levels and cardiac function classification in the experimental group was analyzed. Cardiac Function Classification (NYHA).^14^ Class-I patients with cardiac disease but without resulting limitations of physical activity. Ordinary physical activity does not cause undue fatigue, palpitation, asthma or anginal pain. Class-II patients have cardiac disease and are comfortable at rest. Ordinary physical activity results in undue fatigue, palpitations, asthma or anginal pain, with slight limitation of physical activity. Class-III patients are comfortable at rest. Less than ordinary physical activity causes undue fatigue, palpitations, asthma or anginal pain, with marked limitation of physical activity. Class-IV symptoms of cardiac insufficiency or of anginal syndrome may be present even at rest. Inability to carry on any physical activity without discomfort. Risk factors related to cardiovascular complications were analyzed with cardiovascular complications as the dependent variable, age, hypertension, BMI, Cr, Ca^+2^, PO^-^_4_, GAL-3, Klotho, HDL, LDL and VLDL levels as independent variables, and 0.05 as the significance level of the selected variables.

### Statistical analysis:

All data in this study were statistically analyzed by SPSS 20.0 software, and measurement data were expressed as (*χ̅*±*S*). Two independent sample t-test was used to analyze data between groups, and χ^2^ test was adopted for rate comparison. Logistic regression analysis was utilized to analyze correlation factors, and Pearson correlation coefficient was used to express correlation analysis. P<0.05 indicates a statistically significant difference.

## RESULTS

The levels of Ca^+2^, PO^-^_4_ and GAL-3 in the experimental group were significantly higher than those in the control group, with statistically significant differences (P=0.00), while the level of Klotho in the experimental group was significantly lower than that in the control group, with a statistically significant difference (P=0.00) (Table-II).

**Table-I:** Comparative analysis of the general data between the experimental group and the control group (*χ̅*±*S*) n=40.

Index	Experimental group	Control group	t/χ^2^	P
Age (years)	68.57±6.53	67.72±6.25	0.59	0.55
Gender (Male, %)	27 (%)	23 (%)	0.85	0.36
BMI (kg/m^2^)	27.53±4.66	26.86±4.37	0.66	0.51
Smoking history (cases, %)	21 (%)	23 (%)	0.20	0.65
History of alcoholism (cases, %)	18 (%)	16 (%)	0.20	0.65
History of hyperlipidemia (cases, %)	17 (%)	15 (%)	0.21	0.64
Systolic blood pressure (mmHg)	160.53±26.42	159.14±24.28	0.24	0.81
Diastolic blood pressure (mmHg)	93.84±15.40	95.05±15.21	0.35	0.72

P>0.05.

**Table-II:** Comparative analysis of Ca, P, Gal-3 and Klotho levels between the experimental group and the control group (*χ̅*±*S*) n=40.

Group	Ca^+2^ (mmol/L)*	PO^-^_4_ (mmol/L)*	Gal-3 (μg/L)*	Klotho (pg/ml)*
Experimental group	2.23±0.21	2.18±0.30	10.37±2.06	635.76±203.58
Control group	1.86±0.17	1.83±0.22	7.68±1.83	754.26±186.43
t	8.66	5.92	6.17	2.73
p	0.00	0.00	0.00	0.01

* P<0.05.

The correlation analysis of Ca^+2^, PO^-^_4_, GAL-3, Klotho levels and Cr levels between the two groups suggested that: the levels of Ca and P in the two groups were positively correlated with the level of Cr (P=0.00), the level of Klotho was negatively correlated with that of Cr (P=0.00), but there was no correlation between the level of Gal-3 and the level of Cr (experimental group, P=0.36; control group, P=0.85) (Table-III).

**Table-III:** Correlation analysis between Ca, P, GAL-3, Klotho levels and Cr levels in the two groups (*χ̅*±*S*) n=40.

Group	Experimental group				Control group			

Cr (μmol/L)	Ca^+2^*	PO^-^_4_*	Gal-3	Klotho*	Ca^+2^*	PO^-^_4_*	Gal-3	Klotho*
110~150	0.10	0.21	0.22	-0.62	0.07	0.11	0.17	-0.31
150~200	0.13	0.22	0.20	-0.48	0.14	0.15	0.16	-0.20
>200	0.21	0.26	0.23	-0.23	0.25	0.20	0.17	-0.15
r	2.74	3.97	0.78	5.41	4.83	2.46	0.06	3.07
p	0.00	0.00	0.36	0.00	0.00	0.00	0.85	0.00

* P<0.05.

The correlation analysis between Ca^+2^, PO^-^_4_, GAL-3, Klotho levels and cardiac function in the experimental group suggested that: in the experimental group, the levels of Ca^+2^ and PO^-^_4_ were positively correlated with cardiac function classification (P=0.00), the level of Klotho was negatively correlated with cardiac function classification (P=0.00), while GAL-3 had no significant correlation with cardiac function classification (P=0.76) (Table-IV).

**Table-IV:** Correlation analysis of Ca, P, Gal-3, Klotho levels and cardiac function classification in the experimental group (*χ̅*±*S*) n=40.

Cardiac function classification	Ca^+2^*	PO^-^_4_*	Gal-3	Klotho*
Class I	0.05	0.10	0.16	-0.26
Class II	0.12	0.13	0.15	-0.24
Class III	0.18	0.22	0.16	-0.15
Class IV	0.20	0.27	0.13	-0.11
r	2.03	2.32	0.37	3.08
p	0.00	0.00	0.76	0.00

* P<0.05.

Risk factors related to cardiovascular complications were analyzed with cardiovascular complications as the dependent variable, age, hypertension, BMI, Cr, Ca^+2^, PO^-^_4_, GAL-3, Klotho, HDL, LDL and VLDL levels as independent variables, and 0.05 as the significance level of the selected variables. The results suggested that hypertension, BMI, Cr, Ca^+2^, PO^-^_4_, VLDL and Klotho were all related factors for cardiovascular complications, among which hypertension, BMI, Cr, Ca^+2^, PO^-^_4_ and VLDL and GAL-3 were risk factors (P<0.05), and the level of Klotho was a protective factor (P<0.05) (Table-V).

**Table-V:** Analysis of risk factors related to cardiovascular complications of chronic kidney disease (*χ̅*±*S*).

Observation index	Regression coefficient	OR value	95% CI	P
Age	1.32±0.27	0.73±1.75	(1.44, 0.76)	0.21
Hypertension*	0.15±0.42	1.21±0.30	(1.32, 0.08)	0.00
BMI*	2.53±1.27	4.86±1.44	(3.08, 1.24)	0.00
Cr*	7.35±2.14	8.51±2.38	(8.06, 4.21)	0.00
Ca*	6.41±1.35	7.46±1.07	(7.31, 4.67)	0.00
P*	8.13±1.69	5.62±1.73	(9.24, 6.97)	0.00
Gal-3	3.36±1.23	2.35±0.32	(4.09, 1.06)	0.26
Klotho*	-9.14±2.36	6.18±2.41	(10.24, 6.91)	0.00
HDL	1.38±0.22	0.75±1.72	(1.81, 0.02)	0.32
LDL	3.57±1.28	4.16±0.35	(2.11, 1.73)	0.18
VLDL*	2.11±0.12	1.48±0.23	(2.36, 0.98)	0.02

* P<0.05.

## DISCUSSION

It was confirmed by our results that the level of GAL-3 in patients with cardiovascular complications was significantly higher than those in the control group, with a statistically significant difference (P=0.00), while the level of Klotho was significantly lower than those without cardiovascular complications, with a statistically significant difference (P=0.00). The level of Klotho was negatively correlated with the level of Cr and cardiac function classification (P=0.00). Regression analysis showed that the level of Klotho was a protective factor for cardiovascular complications (P<0.05).

Cardiovascular disease (CVD) is the most common complication and primary cause of death in patients with CKD.^15^ It has been shown in related studies that the risk factors for CVD in patients with CKD include traditional risk factors such as advanced age, smoking, hypertension, abnormal lipid metabolism, diabetes, as well as non-traditional factors such as volume expansion, vascular calcification, calcium and phosphorus metabolism disorder, secondary hyperparathyroidism, malnutrition, elevated homocysteine, and inflammation. Among numerous non-traditional factors, disorder of calcium and phosphorus metabolism is an extremely important one, which is an independent risk factor leading to cardiovascular disease (CVD) and death in patients with CRF.^16^

According to a prospective study of 1203 chronic kidney disease patients before dialysis by Eddington et al.^17^, each one mg/dl increase in serum phosphorus was associated with a 26% increase in mortality and a 50% increase in mortality from cardiovascular events. Since then, calcium and phosphorus metabolism disorders have received more and more attention from scholars.

Abnormal calcium and phosphorus metabolism is a vital mechanism of cardiovascular complications. It was shown in our study that the levels of Ca^+2^ and PO^-^_4_ in patients with cardiovascular complications were significantly higher than those without cardiovascular complications (P=0.00), and the levels of Ca^+2^ and PO^-^_4_ were positively correlated with Cr level and cardiac function classification (P=0.00), suggesting that abnormal calcium and phosphorus metabolism was a risk factor for cardiovascular complications (P<0.05). It has been shown in a study^18^ that vascular and heart valve calcification is the main cause of cardiovascular disease in patients with CRF, with typical pathological changes of calcium and phosphorus deposition in the vascular wall, coronary arteries, heart valves, myocardium, and arterial valves. As a result, complications such as hypertension, congestive heart failure, arrhythmia, ischemic cardiomyopathy and even death may occur. It is currently believed that hyperphosphatemia and hypercalcemia are the primary factors involved in the pathogenesis of cardiovascular disease in patients with CRF.^19^

An increase in blood phosphorus may promote the contraction of vascular smooth muscle cells, and a high phosphorus environment can result in increase binding with existing calcium and acceleration of cell apoptosis. Elevated blood phosphorus and high phosphorus environment together form calcification nests and initiate calcification, which is an important link in vascular calcification caused by hyperphosphatemia.^20^ Hyperphosphatemia promotes smooth muscle extracellular matrix remodeling and enables the appearance of apatite-containing matrix vesicles and calcified collagen fibers on the cell surface. These stromal vesicles will provide sites for further calcification, and stimulation with high phosphorus can increase the secretion of stromal vesicles, thereby aggravating vascular calcification.^21^ According to Vervloet et al. and van Ballegooijen et al.,^22^ high phosphorus can increase the mitochondrial membrane potential and the production of mitochondrial oxygen free radicals, leading to endothelial dysfunction in patients, and eventually causing vascular calcification. In response to this, antioxidant therapy can alleviate the occurrence of vascular calcification in patients with chronic kidney disease.

The DOPPS study has shown a significant increase in cardiovascular mortality in patients with chronic kidney disease when serum calcium levels >2.5mmol/L compared with normal serum calcium levels.^23^ Hypercalcemia can induce calcification of human smooth muscle cells even with normal blood phosphorus levels.^24^ Existing studies have shown that high calcium intake (2 000mg/d) results in positive calcium balance without causing hypercalcemia, suggesting that calcium may be deposited in soft tissues. In this case, blood calcium is further elevated and deposited on the surface of blood vessels. This in turn causes calcification of tissues and blood vessels,^25^ leading to vascular sclerosis and cardiovascular disease.

GAL-3, a member of the galectin-family, is widely expressed in macrophages, neutrophils, eosinophils and mast cells.^26^ These cells play a vital role in the process of myocardial damage, especially in the process of myocardial fibrosis.^27^ It has been shown in a study^28^ that the expression of GAL-3 was significantly increased in the myocardial tissue of mice with fibrosis and hypertrophy in the late stage of hypertension, and GAL-3 inhibitors could be used to intervene in the process of myocardial fibrosis in spontaneously hypertensive rats.

Klotho protein, a unidirectional transmembrane protein expressed in the kidney (main site), heart, brain, parathyroid glands, boasts of promoting renal excretion of phosphorus and inhibiting the synthesis of active vitamin D. It has been demonstrated in a study^29^ that Klotho protein has a protective effect on the kidney in patients with CRF. The deficiency of Klotho protein may cause severe renal damage and fibrosis, and accelerate vascular calcification, which has a close bearing on severe cardiovascular dysfunction.^30^ For patients with advanced CKD, left ventricular hypertrophy is more frequent. Seifert et al.^31^ found that progressive left ventricular hypertrophy also exists in the early stage of CRF, and the decreased expression of Klotho protein would lead to abnormal FGF23 signal transduction, which may be related to progressive left ventricular hypertrophy.

### Limitations:

It includes a small number of patients with a short follow-up time. In future we plan to conduct the study with a large sample size with longer follow up to more objectively assess risk factors, thereby benefiting more patients.

## CONCLUSION

To put it in a nutshell, the levels of Ca^+2^, PO^-^_4_ and GAL-3 in patients with CKD complicated with cardiovascular complications are significantly higher than those in the control group, and the levels of Ca^+2^ and PO^-^_4_ are positively correlated with cardiac function classification. Cr, Ca^+2^ and PO^-^_4_ are risk factors for CKD complicated with cardiovascular disease, while Klotho is a protective factor for cardiovascular disease. Therefore, on the one hand, Ca^+2^, PO^-^_4_ and GAL-3 levels can be used as risk factors for early prediction of cardiovascular complications. On the other hand, certain interventions can be used clinically to reduce Ca^+2^, PO^-^_4_, GAL-3 and increase the content of Klotho, thereby reducing the risk of cardiovascular diseases in patients with CKD.

### Authors’ Contributions:

**ZL and JL** designed this study, prepared this manuscript, are responsible and accountable for the accuracy and integrity of the work.

**QW and XF** collected and analyzed clinical data.

**YG** and **XL** Data analysis, significantly revised this manuscript.
